# Parents of Children with Developmental Difficulties and Parents of Typically Developed Children: What Happens in a Year?

**DOI:** 10.3390/bs10010004

**Published:** 2019-12-18

**Authors:** Lana Lučić

**Affiliations:** Ivo Pilar Institute of Social Sciences, Zagreb 10000, Croatia; lana.lucic@pilar.hr; Tel.: +38514886155

**Keywords:** well-being, parents of children with developmental difficulties, happiness, life events, longitudinal study

## Abstract

Parents of children with developmental difficulties (DD) face many challenges on an everyday basis and, compared to a parent of a typically developed child (TD), are at risk to experience lower well-being. Earlier, as a part of the CRO-WELL project, we explored differences in the well-being of parents of children with DD and a matching group of parents of TD children. Results showed that both groups of parents were equally happy and satisfied with their lives in general, with only a difference in satisfaction with free time. The aim of the current study was to explore what happened in one-year’s time. Out of the initial sample of 41 parents by group, the second wave was completed by 19 parents of DD children and 27 parents of TD children. Results showed that parents of children with DD were less satisfied with life in general, as well as less happy and less satisfied with health, family, friends, and safety compared to parents of TD children. They also experienced three times more negative events than parents of TD children. Having a child with developmental difficulties reflects on many life domains and these results could serve as a guidepost in the design of support for families of children with developmental difficulties. All authors have read and agreed to the published version of the manuscript.

## 1. Introduction

We often hear someone say that parenting is the most important and the hardest job in the world. It brings a lot of joy, but also forces one to change the daily routine, adapt to new life circumstances, and take responsibility for a child that needs to be raised. But when a child is developmentally challenged, parenting is brought to a new level of difficulty and many adjustments must be made to meet various needs of a child.

Early research was focused on negative outcomes of parenting a child with DD, like chronic sorrow [1], depression [2,3], high levels of stress [4,5], increased divorce rates [6], and a lack of free time [7,8]. Many studies showed lower levels of well-being indicators among parents of children with DD when compared to parents of typically developed children [9,10,11]. It was also shown that negative outcomes are related to the severity of the child’s diagnosis, general functioning, and behavior (e.g., [12,13]). For example, parental distress and risk of depression among parents of children with autism were higher compared to both parents of typically developed children and parents of children with a different type of developmental difficulty [12,13,14,15,16]. Other researchers focused more on positive outcomes and found that parenting a child with DD may provide benefits and positive contributions for parents [17] and lead to additional growth and new self-perception [18,19], transformation of beliefs and value systems, priorities and worldview, spiritual experiences, as well as a stronger sense of coherence and control [17,20,21]. Moreover, when these positive outcomes are assessed, differences in well-being between parents of typically or developmentally challenged children is much smaller [22,23,24].

In addition to all the strains that are related to the child’s condition, parents of children with developmental difficulties are not exempt from everyday life and events. Some researchers pointed their attention to exploring how a lack of environmental support, stigma, and social inclusion related to the well-being of parents caring for such a child [25,26,27], showing that negative experiences increase the stress level. However, to the best of our knowledge, life events and its relatedness to the well-being of parents have not yet been studied. Over the last several decades, research on well-being has blossomed, but how major life events relate to subjective well-being could still be considered a mystery. Unlike daily events that continuously form an individual’s life, major life events could be considered as specific transitions. Hopson and Adams [28] define a transition as a discontinuity in life’s routine that a person is aware of, obligating that person to provide a new behavioral response. Life events, positive or negative, characterize everyday life and may influence well-being [29]. Research so far has shown that an individual’s experience of those events might have short- or long-term effects on subjective well-being [30,31]. Short-term effects are easily witnessed, for example after a concert or dinner with friends. On the other hand, the long-term effects of life events on well-being are still unclear. According to the hedonic treadmill or set-point theory, subjective well-being is mostly determined by an individual’s characteristics, and although people may experience a lower or higher level of well-being after some life event, they will in time adapt and return to the initial level [32]. Many researchers confirmed postulates of this theory, e.g., [33,34], but other research showed that some major life events may affect well-being long term e.g., [35,36], such as disability, unemployment, divorce, or death of a spouse. Negative events act as stressors in everyday life and may have a stronger impact on an individual compared to positive events [37,38], while positive events, although linked to positive emotions, were not associated with lower levels of distress [39,40]. Lyubomirsky [40] argued that individuals tend to engage in activities and events that can positively influence their well-being, like getting a pet or starting a new hobby, which might be one of the explanations why the frequency of positive life events contributes to higher levels of positive effects [41]. On the other hand, negative events are usually the result of external circumstances an individual has little control of [39], which might explain their stronger negative influence on well-being compared to positive life events. Additionally, research shows that cumulative adversity (total number of negative events) was associated with lower well-being [42,43], both positive and negative affect [41] while the frequency of positive events was related only to positive affect [41,43]. Some studies were focused on exploring the relationship between life events and PDSD [44], depression or anxiety disorder [45] or risk of developing breast cancer [46], showing the association of life events’ negativity and higher levels of symptoms of disease or higher risk of developing one. Given the life circumstances of parents of children with DD, they may experience a higher frequency of negative life events (e.g., illness, losing a job, financial loss) and lower levels of positive ones (e.g., lack of free time prevents them to have a pet or join a club) which might put additional burden and negatively influence their well-being.

In our previous research [47], we explored differences in well-being between parents who had developmentally challenged (*n* = 41) children and a matching group of parents with typically developed children (*n* = 41). We found that they were equally happy and satisfied with their life in general, but parents of children with DD were less satisfied with their free time. Since the initial study was part of longitudinal research on well-being in Croatia, we were interested to follow up on the first results and explore differences between two groups after one year, as well as to examine possible differences in the occurrence of life events. Given the fact that parenting a child with developmental difficulties is stressful, time demanding, and influential over many aspects of life, we anticipated that well-being of those parents might be lowered compared to parents of typically developed children. Other than satisfaction with free time, we assumed they will feel less satisfied with life in general, happiness, and some other domains of life when compared with parents of typically developed children. We were also interested to see what life events were experienced by two groups of parents in between two-time points. Based on all previous research findings, we anticipated that parents of children with DD would experience higher cumulative adversity and a lower amount of positive events when compared to parents of typically developed children. Additionally, we were interested to see if experienced events, as well as differences between groups, offer a better understanding of well-being in the group of parents of children with developmental difficulties.

## 2. Materials and Methods

The current study was conducted as a part of on-line longitudinal research of well-being in Croatia, open to all adult internet users. Participation in a study was voluntary and anonymous. The research was conducted in 4 waves between 2016 and 2019 and consisted of a comprehensive battery of questionnaires, but only some were used for the purpose of this study:

1. The measure of life satisfaction was assessed by a single-item measure from the World Values Survey [48]. Satisfaction was rated using an 11-point scale, from 0 meaning “not satisfied at all” till 10 “extremely satisfied”.
2. The Happiness Measure Scale [49] was used to assess subjective well-being. Happiness was rated using an 11-point scale, from 0 meaning “not happy at all” till 10 “extremely happy”.
3. The Personal Well-being Index [50] was used to assess satisfaction with various life domains. The scale consists of seven items rated on an 11-point scale (0–10): standard of living, health, achievement in life, relationships, safety, community connectedness, and future security. For the purpose of the longitudinal research, we adapted PWI by dividing “relationships” into two categories (family and friends) and by adding four additional domains: free time, work, physical appearance, and love life. In the initial sample of this study, the Cronbach’s α of the adapted personal well-being index was 0.88.
4. A Life Events Scale was designed for the purpose of the longitudinal research based on the list of life events by Leist et al. [51] and Ballas and Dorling [52]. It consisted of 69 events divided into five categories: love, family and home; job and finance; health; leisure time; and legal system. Each participant was asked to mark all the events that have happened in the previous year and then provide an estimation of how positive and negative each of those events was for him/her using an 11-point scale (0–10). Based on the estimation of positivity and negativity of all events, using the entire sample of the CRO-WELL longitudinal research, events were later divided into positive (27 events) and negative (27 events), while the remaining 15 events were excluded from further analysis as ambiguous.

Using the total sample of the longitudinal study, results from the first wave were used to conduct the initial study [47]. A sample consisted of 41 parents of children with developmental difficulties and 41 parents of typically developed children, matched using many demographic factors. In 2017, the second wave was completed by 19 parents of children with DD and 27 parents of typically developed children from the initial sample and their results were used for the purpose of currents study. As is visible in Table 1, the two groups of parents remained to be similar regarding demographic characteristics: In both groups, the majority of the participants were women, married, employed, with a higher education, and a similar monthly income.

## 3. Results

Due to the small sample size, all analyses were conducted using non-parametric statistics. Since multiple comparisons were made, a Bonferroni correction was applied. All analyses were conducted using SPSS 24 (IBM, Chicago, IL, USA).

### 3.1. Overall Life Satisfaction and Happiness

Overall life satisfaction and happiness were assessed as two general indicators of well-being (Table 2). Results showed that parents of children with developmental difficulties were less satisfied with their lives and reported a lower level of happiness. These results were in contrast with results from the initial study [47] where no significant difference was found between the two groups of parents in general well-being indicators.

### 3.2. Satisfaction With Various Life Domains

Regarding satisfaction with various life domains, parents of children with developmental difficulties were less satisfied with health, relationships with family and friends, safety, and future security (Table 3). In our initial study, the only difference between the two groups of parents was found in satisfaction with free time, which was not significant in this study.

### 3.3. Life Events

Parents of children with DD reported 127 experienced life events, out of which 76 were positive and 34 negative. Parents of TD children experienced a total of 182 life events, of which 137 were positive and 17 negative. On average, parents of children with DD experienced a similar amount of all life events in the previous year, but less positive—almost three times more negative events per person (Table 4.)

Closer examination of events revealed further differences. In the top 10 life events, parents of children with disabilities experienced 3 negative life events (illness of a close one, failure of a child at school, and financial loss) while all top 10 events for parents of typically developed children were positive ones (Table 5). Even more, none of the parents of children with DD was promoted at work in the previous year, only one got a job, and only three reported health improvement. Although small in a number of occurrences, some negative events were experienced only by parents of children with DD like diagnosed personal illness, serious injury, and a threat or house robbery.

## 4. Discussion

A group of parents of children with developmental difficulties were identified during the first stage of the longitudinal research. They were matched to a group of parents of typically developed children using many socio-demographic criteria and possible differences in well-being were tested. The sample mostly consisted of educated, employed middle-class women and the only difference between the two groups was in satisfaction with free time. This study aimed to explore the differences between the two groups after one year. As hypothesized, differences in well-being between the two groups of parents emerged, indicating parents of children with DD were less happy and satisfied with their lives, health, and relationships with family and friends. On the other hand, the two groups of parents expressed the same level of satisfaction with free time. As expected, parents of children with DD experienced more negative and less positive life events compared to another group of parents. Regarding socio-demographic characteristics, the two groups remained quite similar, despite the dropout.

Results of this study indicate that the flow of time brought certain changes causing differences in well-being between groups to emerge. In line with previous research, parents of children with DD appeared to have lower well-being compared to another group of parents [9,10,53]. Daily struggles and often having a very busy schedule contribute to the deterioration of health, not only due to stress but also as a result of a parent’s postponement of care for personal health. It was shown that parents of children with DD suffer from many health problems [54,55] and all of that may contribute to a lower level of satisfaction with personal health. Many researches so far have pointed out the relevance of support from family and society, e.g., [25,26,27], indicating that good relationships serve as a stress protector. In some cases, support is denied because of a lack of understanding, but another possible explanation for poorer satisfaction with relationships might be found in life circumstances of parents of children with DD. Many of them devote themselves entirely to the care of a child [8], neglecting social activities [56,57], which may lead to lower satisfaction with relationships that they formed with family and friends. Contrary to expectations, two groups of parents were equally satisfied with free time. However, the reasons behind a lowered well-being may also influence the perception of free time, making them feel satisfied with any available moment they can spare for themselves. Life circumstances, as well as a constant battle with the system, may influence a parent’s sense of security, especially in terms of the future as they are additionally burdened with worries from the child’s perspective [58], leading to poorer satisfaction with both current and future security.

Regarding life events, parents of children with DD experienced nearly three times more negative events per person compared to another group of parents, which could also contribute to a lower level of their well-being [41,43]. Three negative events that appeared in the top 10 of the most frequent ones in the group of parents of children with DD, could all be associated with their life circumstances. For example, a child’s or spouse’s health might have deteriorated and an unexpected cost of care emerged. Additionally, the school system is not adjusted to the needs of children with DD and often they lack the support needed to fully engage and execute given tasks, causing them to fail. The difference in frequency of journeys is also understandable. Although both groups of parents go on vacation, parents of typically developed children travel much more. Other than financial strain, it is possible that planning a trip, as well as going on one, might be very complicated in families with developmentally challenged children due to the child’s condition. Differences regarding job and career confirm research findings showing that parents of children with DD are either limited to take part in paid work or often forced to quit in order to take care of the child [54,55]. Considering the set-point theory, experienced life events may contribute to lower well-being of parents of children with DD. However, the theory also postulates that the level of well-being returns to the starting point after some period of adaptation. Therefore, the lack of differences between the two groups of parents one year ago [47] may have been a result of some major events that happened in the year before. Those events may have resulted in a positive effect on the well-being of parents of children with DD, causing differences between two groups of parents to diminish. In that case, emerged differences in time may be a result of an adaptation process and return of well-being of parents of children with DD to the set-point. 

As the sample size was reduced, there was a possibility that these findings are a result of sample characteristics. Emerged differences might happen because follow-up was completed by less happy and satisfied parents of children with DD or more satisfied and happy parents of typically developed children. First, the possible change in well-being indicators between the two time points in each group was examined and no differences were found: Both groups of parents remained at the same level on all indicators after one year. This finding proved that differences should be attributed to the sample, and not to the differences between the two time points. Then, two groups of parents were compared in Time 1. The parents of children with DD that participated in the follow-up study were less satisfied with their life (U = 149, *p* = 0.014), less happy (U = 129, *p* = 0.004), and had lower satisfaction with health (U = 112.5, *p* = 0.001) and free time (U = 112.5, *p* = 0.001) in Time 1 compared to group of parents of TD children that also participated in follow-up. At last, well-being indicators in Time 1 of both groups of parents were compared with those who failed to participate again. There was no statistical difference between the well-being of parents of children with DD who continued the survey and those who did not and the same was with parents of TD children, although closer inspection of data revealed there was a trend among parents of typically developed children, suggesting that those who were happier remained in research. A similar trend was not found among parents of children with disability. In the initial research, the group of parents of typically developed children was selected to match the characteristics of the group of parents with children with developmental difficulties. Although the drop off was bigger in the group of parents of children with DD (about half dropped out compared to about 1/3 of parents of typically developed children), it seems that drop off was systematical only in the latter group where those with lower well-being tended to drop off more often. This trend among parents of typically developed children is most probably due to the small sample size where any drop-off can systematically influence results. 

The biggest limitation of this study was a small, self-selected sample which restricted data analysis and generalization. Future research should aim to eliminate this limitation. A larger and less convenient sample should be obtained. One should also consider implementing some other scales that might give better insight into the positive outcomes of parenting a child with DD, like flourishing. Finally, implementing a longitudinal research design that would address all aspects used in this study, and the proposed changes, might significantly contribute to a better understanding of life with a developmentally challenged child and serve as a solid ground to build a support system.

## Figures and Tables

**Table 1:** Socio-demographic characteristics of parents of children with developmental disability and parents of typically developed children.

Feature		Parents	
		DD	TD
*n*		19	27
Gender (F)		17	23
Married		16	26
Employed		16	22
Education	≤high school	7	10
	>high school	12	17
Monthly income	<2.000 HRK	2	7
	2.000–5.000 HRK	15	18
	≥5.001 HRK	2	2

Note: DD—developmental disability; TD—typically developed.

**Table 2:** Results of testing differences in overall life satisfaction and happiness between parents of children with developmental difficulties and parents of typically developed children.

Well-Being Indicator	Parents	Mode	Mdn	Mean Rank	Mann–Whitney U	Wilcoxon W	z Value	*p*
Life satisfaction	DD	6	5	17.63	145	335	−2.52	0.01 *
	TD	7	8	27.63				
Happiness	DD	6	5	16.18	117.5	307.5	−3.16	0.001 *
	TD	7	7	28.65				

Note: DD—developmental difficulties; TD—typically developed; Mdn—median.

**Table 3:** Results of testing differences in satisfaction with various life domains between parents of children with developmental difficulties and parents of typically developed children.

Satisfaction with	Parents	Mode	Mdn	Mean Rank	Mann–Whitney U	Wilcoxon W	z Value	*p*
Standard of living	DD	5	6	21.53	219	409	−0.85	0.202
	TD	5	6	24.89				
Health	DD	8	6	15.37	102	292	−3.51	0.000 *
	TD	9	8	29.22				
Life achievement	DD	7	7	22.00	228	418	−0.65	0.263
	TD	9	7	24.56				
Relationship with family	DD	6	7	16.89	131	321	−2.88	0.002 *
	TD	9	9	28.15				
Relationship with friends	DD	6	7	16.68	127	317	−2.94	0.001 *
	TD	9	9	28.30				
Safety	DD	5	5	16.08	115.5	305.5	−3.18	0.001 *
	TD	8	8	28.72				
Community connectedness	DD	5	6	18.26	157	347	−2.26	0.012
	TD	8	8	27.19				
Future security	DD	2	3	17.39	140.5	330.5	−2.61	0.004 *
	TD	7	6	27.80				
Free time	DD	7	4	18.45	160.5	350.5	−2.16	0.015
	TD	9	7	27.06				
Work	DD	8	7	22.42	236	426	−0.46	0.325
	TD	8	7	24.26				
Physical appearance	DD	9	6	20.11	192	382	−1.46	0.074
	TD	7	7	25.89				
Love life	DD	5	7	19.47	180	370	−1.73	0.044
	TD	10	8	26.33				

Notes: DD—developmental difficulties; TD—typically developed; Mdn—median; Bonferroni’s corrected * *p* < 0.004.

**Table 4:** The average number of experienced life events in the previous year between parents of children with developmental difficulties and parents of typically developed children.

Life Events	Parents	
	DD	TD
All LE/per person	6.68	6.74
Positive LE/per person	4	5.07
Negative LE/per person	1.79	0.63

Notes: LE—life events; DD—children with developmental difficulties; TD—typically developed.

**Table 5:** Top 10 of the most common life events experienced by the two groups of parents in the previous year.

Parents of Children with DD (F)	Top 10	Parents of TD Children (F)
Vacation (12)	1	Vacation (18)
Success of a child at school (10)	2	Success of a child at school (17)
Friendship (8)	3	Journey (17)
Illness of close one (7)	4	Friendship (14)
New activity (7)	5	Health improvement (10)
Joining a club (7)	6	Acknowledgement at work (9)
Journey (7)	7	New activity (9)
Volunteer work (7)	8	Getting a job (8)
Failure of a child at school (6)	9	Big buy (7)
Financial loss (5)	10	Promotion at work (5)

Notes: DD—developmental difficulties; TD—typically developed; F—frequency.

## References

[1] Olshansky S. (1962). Chronic sorrow: A response to having a mentally defective child. Soc. Case-Work.

[2] Quine L., Pahl J. (1985). Examining the causes of stress in families with severely mentally handicapped children. Br. J. Soc. Work.

[3] Roach M.A., Orsmond G.I., Barratt M.S. (1999). Mothers and fathers of children with Down syndrome: Parental stress and involvement in childcare. Am. J. Ment. Retard..

[4] Hastings R.P., Beck A. (2004). Practitioner review: Stress intervention for parents of children with intellectual disabilities. J. Child Psychol. Psychiatry.

[5] Singer G.H. (2006). Meta-analysis of comparative studies of depression in mothers of children with and without developmental disabilities. Am. J. Ment. Retard..

[6] Gath A. (1977). The impact of an abnormal child upon the parents. Br. J. Psychiatry.

[7] Barnett W.S., Boyce G.C. (1995). Effects of children with Down syndrome on parents’ activities. Am. J. Ment. Retard..

[8] Sanders J., Morgan S. (1997). Family Stress and Adjustment as Perceived by Parents of Children with Autism or Down Syndrome: Implications for Intervention. Child Fam. Behav. Ther..

[9] Byrne M.B., Hurley D.A., Daly L., Cunningham C.G. (2010). Health status of caregivers of children with cerebral palsy. Child Care Health Dev..

[10] Guyard A., Fauconnier J., Mermet M.A., Cans C. (2011). Impact on parents of cerebral palsy in children: A literature review. Arch. Pediatrie.

[11] Baker B.L., Blacher J., Crnic K., Edelbrock C. (2002). Behavior problems and parenting stress in families of three-year old children with and without developmental disabilities. Am. J. Ment. Retard..

[12] Hastings R.P. (2003). Child behaviour problems and partner mental health as correlates of stress in mothers and fathers of children with autism. J. Intellect. Disabil. Res..

[13] Cuzzocrea F., Larcan R., Baiocco R., Costa S. (2011). Family functioning, parenting, and couple satisfaction in families of children with a disability. Disabil. Family.

[14] Dunn M.E., Burbine T., Bowers C.A., Tantleff-Dunn S. (2001). Moderators of stress in parents of children with autism. Community Ment. Health J..

[15] Larcan R., Cuzzocrea F. (2011). Funzionamento della famiglia e sviluppo psico-sociale dei fra-telli di individui con disabilità intellettive. Psicologia Clinica dello Sviluppo.

[16] Hastings R.P., Taunt H.M. (2002). Positive perceptions in families of children with developmental disabilities. Am. J. Ment. Retard..

[17] Tedeschi R.G., Calhoun L.G. (2004). Posttraumatic growth: Conceptual foundations and empirical evidence. Psychol. Inq..

[18] Tedeschi R.G., Tedeschi R.G., Park C.L., Calhoun L.G. (1998). Posttraumatic Growth: Positive Changes in the Aftermath of Crisis.

[19] King G.A., Zwaigenbaum L., King S., Baxter D., Rosenbaum P., Bates A. (2006). A qualitative investigation of changes in the belief systems of families of children with autism or Down syndrome. Child Care Health Dev..

[20] Gray D.E. (2006). Coping over time: The parents of children with autism. J. Intellect. Disabil. Res..

[21] Affleck G., Tennen H., Gershman K. (1985). Cognitive adaptations to high-risk infants: The search for mastery, meaning, and protection from future harm. Am. J. Ment. Defic..

[22] Abbott D., Meredith W. (1986). Strengths of Parents with Retarded Children. Family Relat..

[23] Scorgie K., Sobsey D. (2000). Transformational outcomes associated with parenting children who have disabilities. Ment. Retard..

[24] Green S.E. (2003). “What do you mean ‘what’s wrong with her?’”: Stigma and the lives of families of children with disabilities. Soc. Sci. Med..

[25] Resch J.A., Mireles G., Benz M.R., Grenwelge C., Peterson R., Zhang D. (2010). Giving parents a voice: A qualitative study of the challenges experienced by parents of children with disabilities. Rehabil. Psychol..

[26] Woodgate R.L., Ateah C., Secco L. (2008). Living in a world of our own: The experience of parents who have a child with autism. Qual. Health Res..

[27] Goodyer I.M. (1996). Recent undesirable life events: Their influence on subsequent psychopathology. Eur. Child Adolesc. Psychiatry.

[28] Hopson B., Adams J., Adams J., Hayes J., Hopson B. (1976). Towards an understanding of transition: Defining some boundaries of transition dynamics. Transition: Understanding and Managing Personal Change.

[29] Luhmann M., Hofmann W., Eid M., Lucas R.E. (2012). Subjective well-being and adaptation to life events: A meta-analysis. J. Personal. Soc. Psychol..

[30] Hentschel S., Eid M., Kutscher T. (2017). The influence of major life events and personality traits on the stability of affective well-being. J. Happiness Stud..

[31] Larsen J.T., McGraw A.P., Cacioppo J.T. (2001). Can people feel happy and sad at the same time?. J. Personal. Soc. Psychol..

[32] Headey B. (2008). Life Goals Matter to Happiness: A Revision of Set-Point Theory. Soc. Indic. Res..

[33] Wildeman C., Turney K., Schnittker J. (2014). The hedonic consequences of punishment revisited. J. Crim. Law Criminol..

[34] Lucas R.E., Clark A.E., Georgellis Y., Diener E. (2003). Reexamining adaptation and the set point model of happiness: Reactions to changes in marital status. J. Personal. Soc. Psychol..

[35] Lucas R.E. (2007). Adaptation and the set-point model of subjective well-being: Does happiness change after major life events?. Curr. Dir. Psychol. Sci..

[36] Diener E., Lucas R.E., Scollon C.N. (2006). Beyond the hedonic treadmill: Revising the adaptation theory of well-being. Am. Psychol..

[37] Baumeister R.F., Bratslavsky E., Finkenauer C., Vohs K.D. (2001). Bad is stronger than good. Rev. Gen. Psychol..

[38] Zautra A.J., Reich J.W. (1983). Life events and perceptions of life quality: Developments in a two-factor approach. J. Community Psychol..

[39] Zautra A.J., Affleck G.G., Tennen H., Reich J.W., Davis M.C. (2005). Dynamic approaches to emotions and stress in everyday life: Bolger and Zuckerman reloaded with positive as well as negative affects. J. Personal..

[40] Lyubomirsky S., Sheldon K.M., Schkade D. (2005). Pursuing happiness: The architecture of sustainable change. Rev. Gen. Psychol..

[41] Prizmić-Larsen Z., Kaliterna-Lipovčan L., Larsen R., Brkljačić T., Brajša-Žganec A. (2019). The Role of Flourishing in Relationship between Positive and Negative Life Events and Affective Well-Being. Appl. Res. Qual. Life.

[42] Seery M.D., Holman E.A., Silver R.C. (2010). Whatever does not kill us: Cumulative lifetime adversity, vulnerability, and resilience. J. Personal. Soc. Psychol..

[43] Burns R.A., Machin M.A. (2013). Psychological wellbeing and the diathesis-stress hypothesis model: The role of psychological functioning and quality of relations in promoting subjective well-being in a life events study. Personal. Individ. Differ..

[44] Sundin E.C., Horowitz M.J. (2003). Horowitz’s impact of event scale evaluation of 20 years of use. Psychosom. Med..

[45] Espejo E.P., Hammen C., Brennan P.A. (2012). Elevated appraisals of the negative impact of naturally occurring life events: A risk factor for depressive and anxiety disorders. J. Abnorm. Child Psychol..

[46] Fischer A., Ziogas A., Anton-Culver H. (2018). Perception matters: Stressful life events increase breast cancer risk. J. Psychosom. Res..

[47] Lučić L., Brkljačić T., Kaliterna Lipovčan L. (2017). A comparison of well-being indicators and affect regulation strategies between parents of children with disabilities and parents of typically developed children. Hrvat. Rev. Rehabil. Istraživanja.

[48] World Values Survey. www.worldvaluessurvey.org.

[49] Fordyce M.W. (1988). A review of research on The Happiness Measures: A sixty second index of happiness and mental health. Soc. Indic. Res..

[50] Cummins R.A. (1996). The Domains of Life Satisfaction: An Attempt to Order Chaos. Soc. Indic. Res..

[51] Leist A.K., Ferring D., Filipp S.-H. (2010). Remembering positive and negative life events: Associations with future time perspective and functions of autobiographical memory. GeroPsych–J. Gerontopsychol. Geriatr. Psychiatry.

[52] Ballas D., Dorling D. (2007). Measuring the impact of major life events upon happiness. Int. J. Epidemiol..

[53] Ergün S., Ertem G. (2012). Difficulties of mothers living with mentally disabled children. Development.

[54] Witt W.P., Riley A.W., Coiro M.J. (2003). Childhood functional status, family stressors, and psychosocial adjustment among school-aged children with disabilities in the United States. Arch. Pediatrics Adolesc. Med..

[55] Wolfensberger W., Baumeister A.A. (1969). Counseling the parents of the retarded. Mental Retardation: Appraisal, Education, and Rehabilitation.

[56] Shearn J., Todd S. (2000). Maternal employment and family responsibilities: The perspectives of mothers of children with intellectual disabilities. J. Appl. Res. Intellect. Disabil..

[57] Seltzer M.M., Greenberg J.S., Floyd F.J., Pettee Y., Hong J. (2001). Life course impacts of parenting a child with a disability. Am. J. Ment. Retard..

[58] Heiman T. (2002). Parents of children with disabilities: Resilience, coping, and future expectations. J. Dev. Phys. Disabil..

